# Long segment 3D double inversion recovery (DIR) hypersignal on MRI in glaucomatous optic neuropathy

**DOI:** 10.1186/s12886-019-1273-0

**Published:** 2019-12-16

**Authors:** Thomas Sartoretti, Jörg Stürmer, Elisabeth Sartoretti, Arash Najafi, Árpád Schwenk, Michael Wyss, Christoph Binkert, Sabine Sartoretti-Schefer

**Affiliations:** 1 Laboratory of Translational Nutrition Biology, Department of Health Sciences and Technology, 8603 ETH Zürich, Schwerzenbach Switzerland; 2 Department of Ophthalmology, Cantonal Hospital Winterthur, Brauerstrasse 15, 8401 Winterthur, Switzerland; 3 Department of Radiology, Cantonal Hospital Winterthur, Brauerstrasse 15, 8401 Winterthur, Switzerland; 4 Faculty of Medicine, University of Zürich, Zürich, Switzerland; 5 Philips Health Systems, Zürich, Switzerland

**Keywords:** Glaucoma, Glaucomatous optic neuropathy, Double inversion recovery (DIR) MR sequence, DIR hypersignal, Optical coherence tomography (OCT)

## Abstract

**Background:**

In this retrospective study the relationship between intraocular pressure (IOP), retinal nerve fiber layer (RNFL) thickness and pathologic hypersignal in optic nerve segments on 3D double inversion recovery (DIR) MR sequence in 21 patients with proven glaucoma of different origin was evaluated.

**Methods:**

All patients were examined on a 3 T MR Philips® scanner. Pathologic optic nerve DIR hypersignal was determined in four different nerve segments. IOP was measured in mmHg by applanation tonometry. RNFL thickness was measured in μm with optical coherence tomography (OCT Heidelberg Engineering Spectralis® apparatus). Wilcoxon rank sum tests, student’s t-tests and (multivariate) linear regression models were appied.

**Results:**

3D DIR hypersignal was present in 17 (41.5%) optic nerves. 3D DIR hypersignal was not related to ischemic or demyelinating optic nerve pathology but was associated with increased IOP (19.8 [24–18]; versus 15.45; [18.85–13.75] mmHg; *p* = 0.008) and decreased RNFL thickness (61.06 ± 12.1 versus 82.5 ± 21.6 μm; *p* < 0.001) in comparison to optic nerves of glaucoma patients without DIR hypersignal. Specifically, presence of DIR hypersignal in optic nerves in at least one optic nerve segment lowered RNFL thickness on average by 17.54 μm (*p* = 0.005) in comparison to optic nerves without DIR hypersignal.

**Conclusions:**

In patients with glaucomatous optic neuropathy (GON) and pathologic optic nerve DIR hypersignal, significantly increased IOP and significantly decreased RNFL thickness values are present. DIR hypersignal seems to be a marker for disease severity in GON related to decreased RNFL thickness and may thus represent long-segment severe axonal degeneration in optic nerves in patients with GON. Venous congestion and edema within the optic nerve related to high IOP may contribute to the DIR hypersignal as well.

## Background

Glaucoma comprises a group of irreversible and progressive eye diseases that result in damage to the optic nerve [1–3] due to decay of retinal ganglion cells (RGCs) and degeneration of their axons [4–7].The damage to the optic nerve is caused by mechanical and vascular factors together with impairment arising by compartimentalization of the cerebrospinal fluid around the optic nerve [8–14] whereas the intraocular pressure (IOP) is the main critical causative risk factor [8]. The neural damage is enhanced by additional risk factors as genetic factors, increased patient age, family history of glaucoma, thin corneal thickness, low ocular systolic / diastolic pressure or low mean ocular perfusion pressure and obstructive sleep apnoea syndrome [9–13].

The damage to the RGCs and axons is confirmed by thinning of the peripapillary retinal nerve fiber layer (RNFL) thickness that is quantitatively measurable by optical coherence tomography (OCT) [15–19].

Usually glaucoma is diagnosed by clinical and ophthalmologic examinations (examination of the visual field, measurements of the intraocular pressure, RNFL thickness measurements etc.) and MRI of the optic system is not considered necessary for diagnosis. In patients with atypical and asymmetric visual field defects, however, where the diagnosis of glaucoma is not easily achieved, an additional MR examination of brain and orbits is added to exclude ischemic and demyelinating pathology [20–23].

In several of these patients unexpectedly pathologic long segment 3D Double Inversion Recovery (3D DIR) hypersignal of multiple optic nerve segments was observed that was not related to ischemic or demyelinating optic nerve pathology. Thus, in our retrospective study we wondered if there was a relationship between IOP and RNFL thickness values (representing disease severity) and pathologic DIR hypersignal in these patients.

## Methods

### Patient selection

21 glaucoma patients (18 open-angle glaucoma, 2 angle-closure glaucoma, 1 normal tension glaucoma, either uni- or bilateral presentation) who were given a dedicated MRI examination of brain and orbits were identified (41 eyes were included in the analysis). Patients (10 males, 11 females) had a mean age of 64 years (meals: 51–80 years; females: 45–83 years). All patients used 1 to 4 different drugs for IOP control. Glaucoma was diagnosed based on ophthalmologic examinations, on the average intraocular pressure (IOP) with 2–4 pressure measurements performed by applanation tonometry within 1 month before MRI and with a pathologic IOP of more than 21 mmHg, on OCT measurements and on visual field measurements (OCTOPUS perimetry using dynamic strategy G2 program with analysis of mean deficit or Goldman perimetry) [19].

Atypical and asymmetric visual field defects, not in accordance with the classical appearance of a glaucomatous optic papilla on fundoscopy, resulted in the decision of the ophthalmologist to perform MRI examination in all these patients in order to exclude additional vascular and demyelinating orbital or cerebral pathology. Toxic, metabolic, nutritional and hereditary optic neuropathy were excluded by laboratory and clinical evaluation.

### Ophthalmologic examination

The thickness of the peripapillary RNFL in 6 sectors at the optic nerve head (ONH) was determined by OCT on a Heidelberg Engineering Spectralis® apparatus and the arithmetic mean determined the resulting RNFL thickness value [19]. Highly pathologic values correspond to a RNFL thickness of 40 to 76 μm, pathologic values to a thickness of 77 to 85 μm and normal values to a thickness of over 86 μm (data supplied by Heidelberg Engineering®). OCT measurements were obtained within 2 months before or after the MRI examination.

### MRI examination

All MRI examinations were obtained on a 3 T MR Achieva Philips® scanner. Beside other sequences as 3D fluid attenuated inversion recovery (FLAIR), T2 weighted (w) turbo spin echo (TSE) and diffusion weighted (DWI) sequences a sagittal 3D DIR sequence (sequence parameters depicted in Table 1) with reconstruction of coronal and transverse images (slice thickness of 1 mm) was acquired and these sequences helped to exclude ischemic disease. Periventricular, juxtacortical and infratentorial lesions were absent on 3D FLAIR and on 3D DIR [20–22] and thus demyelinating disease could be excluded based on McDonald criteria for the diagnosis of multiple sclerosis [22]. Ischemic optic neuropathy could be excluded because of missing diffusion restriction on DWI and because of missing history of acute vision loss.

**Table 1:** Imaging parameters of the 3D DIR sequence on 3 T MR Achieva® Philips

	3D double inversion recovery DIR
Acquisition mode	3D turbo spin echo
Acquisition plane	sagittal
Coverage	whole head
Reconstructions, slice thickness	coronal, 2 mm
TR / TE	5500 ms/ 246 ms
TI	2550 ms / 450 ms
FOV	250x250x195 mm
Matrix	240x240x310
Acquired voxel size	1.2 × 1.2 × 0.65 mm
Number of slices	300
Fat suppression	spectral presaturation with inversion recovery SPIR
Number of excitations	2
Acquisition time	6 min 19 s.

Pathologic DIR hypersignal of optic nerve segments (i.e. retrobulbar, canalicular, prechiasmatic, chiasmatic segments) was rated in comparison with the signal intensity of the ipsilateral lateral rectus muscle [23]. Long-segment DIR hypersignal was defined as hypersignal in at least 2 nerve segments. Pathologic DIR hypersignal was confirmed on corresponding coronal T2w STIR images or on the T2w m-Dixon TSE sequence. Rating was performed in consensus by two board certified neuroradiologist with 30 years and 5 years of experience.

### Statistical analysis

The Kolmogorov-Smirnov test was used to check normality of data. Wilcoxon rank sum tests or student’s t-tests and (multivariate) linear regression models were then applied to test the relationship between hypersignal, RNFL thickness and IOP. Age was included in the regression models to account for a physiological decrease in RNFL thickness with increasing age. Significance was set at *p* ≤ 0.05. Data is presented either as mean ± standard deviation (SD) in case of normally distributed data or median;[interquartile range (IQR)] in case of non-normally distributed data. For statistical analysis IBM SPSS Statistics, Version 25.0 was used.

## Results

**Fig. 1:**
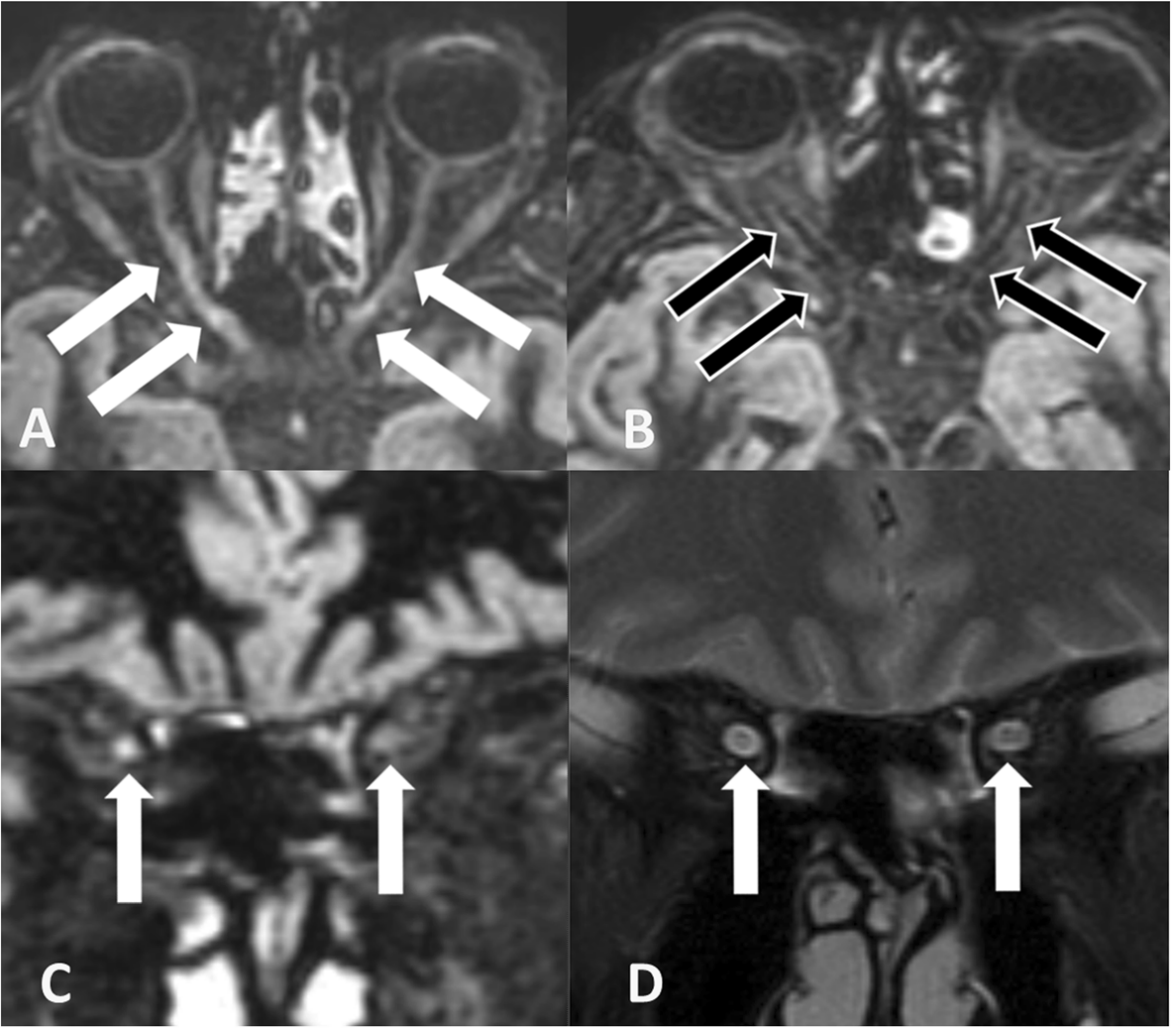
Bilateral DIR hypersignal in retrobulbar and canalicular optic nerve segments (white arrows on the transverse image reconstruction in **a** and on the coronal image reconstruction in **c**) compared to normal DIR hypointense retrobulbar and canalicular optic nerve segments (black arrows with white rim on transverse image reconstructions in **b**). For comparison hypersignal in the retrobulbar segment is also shown on the coronal T2w m-Dixon turbo spin echo image (white arrows in **d**)

**Fig. 2:**
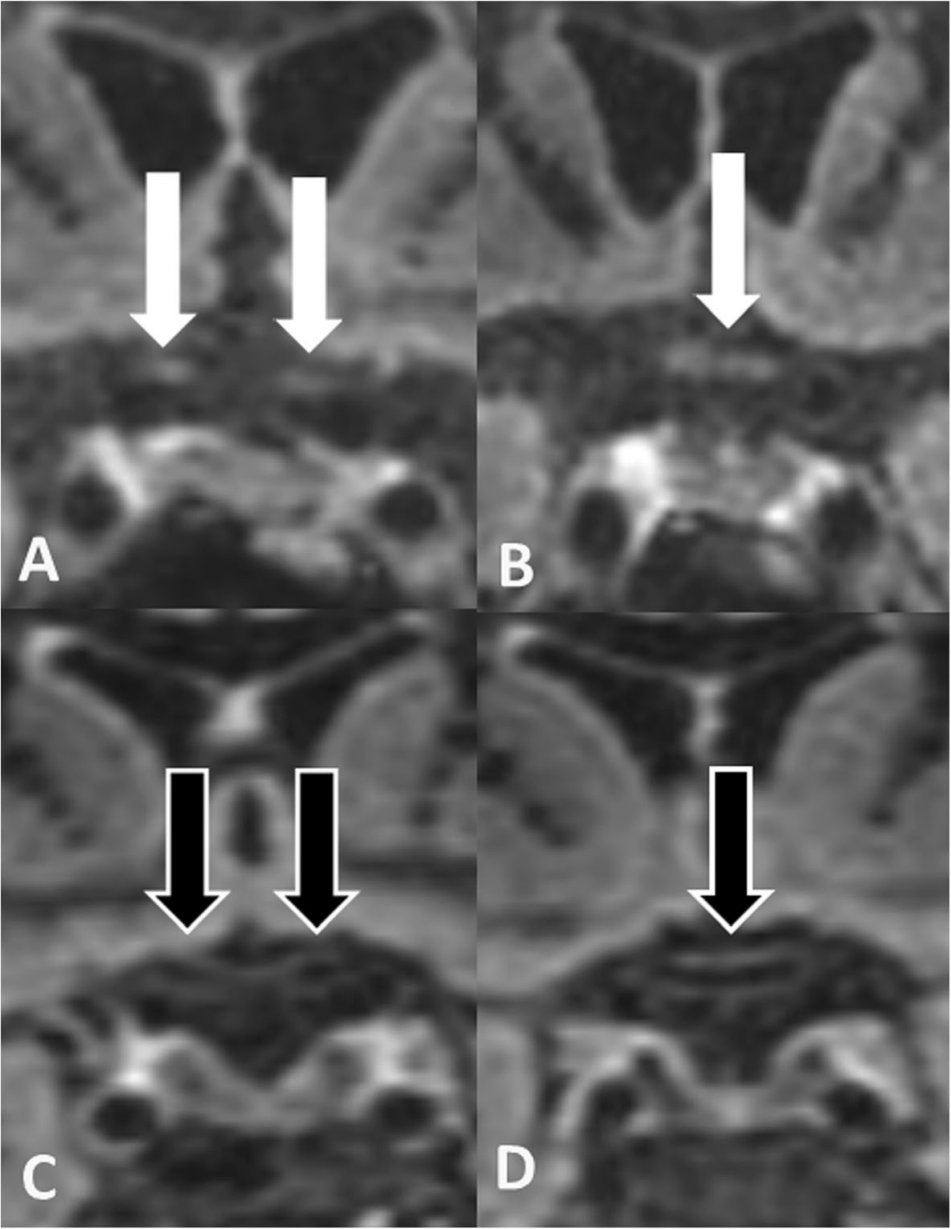
Coronal DIR reconstructions presenting with bilateral DIR hypersignal in prechiasmatic (**a**) and chiasmatic (**b**) optic nerve segments (white arrows) compared to normal DIR hypointense prechiasmatic (**c**) and chiasmatic (**d**) optic nerve segments (black arrows with white rims)

In 17 (41.5%) of 41 optic nerves DIR hypersignal in at least one optic nerve segment was present (Figs. 1 and 2). DIR hypersignal was observed in 1 nerve segment in 5.9%, in 2 segments in 35.3%, in 3 segments in 52.9% and in 4 segments in 5.9% of these 17 nerves as shown in Fig. and Fig. 2. Thus long-segment DIR hypersignal was present in 94.1% of DIR hyperintense optic nerves.

There was no significant relationship between the number of segments displaying hypersignal and IOP or RNFL thickness values.

**Fig. 3:**
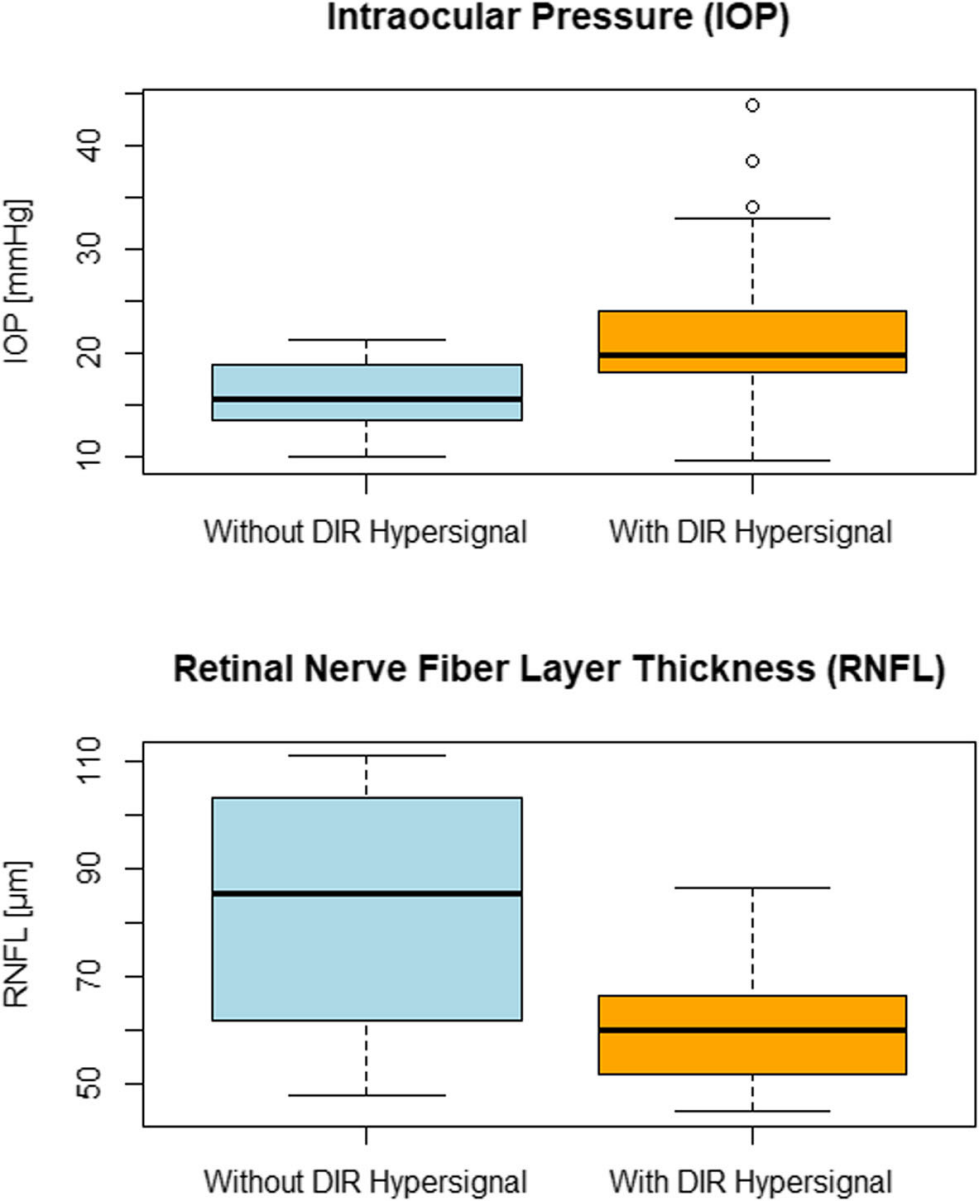
Upper boxplot diagram depicting intraocular pressure data. Lower boxplot diagram depicting RNFL thickness data The median is depicted as a prominent line in the middle of the box, the mean as a cross and 0.75 respectively 0.25 quantiles as the upper and lower limits of the box.

**Fig. 4:**
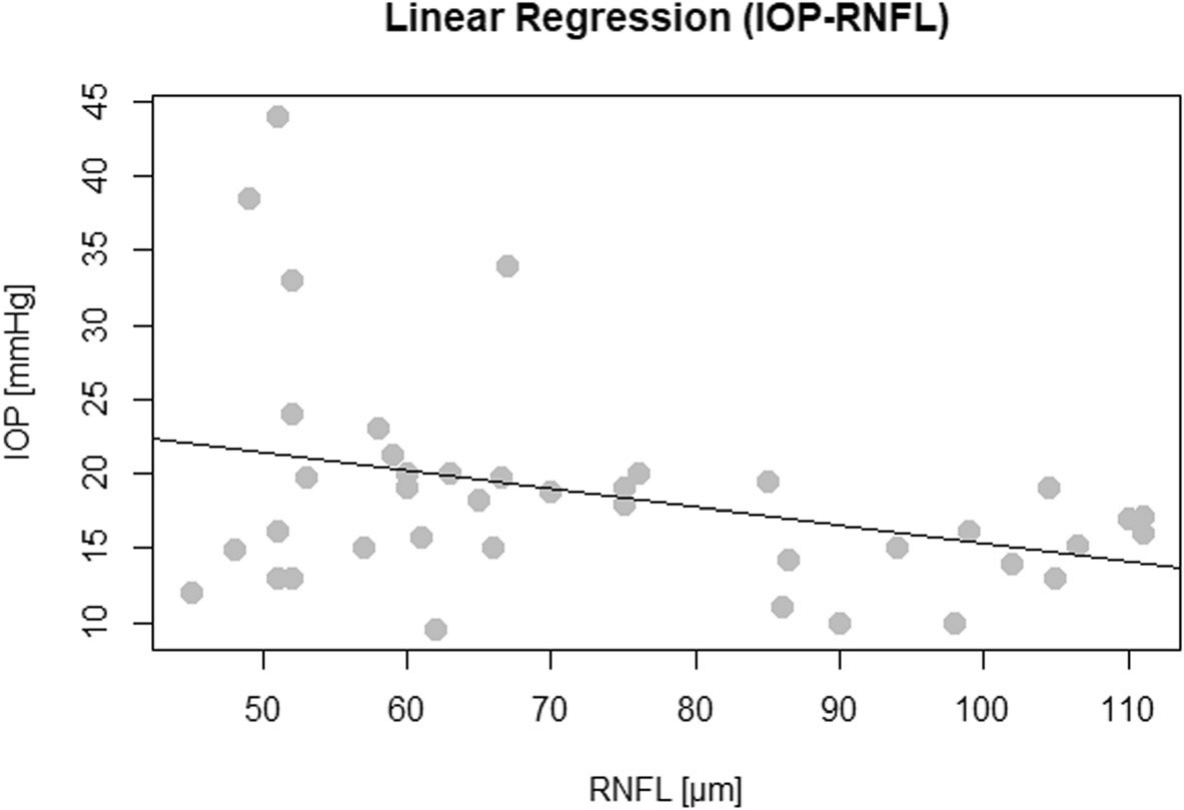
Linear regression model depicting the linear relationship between IOP values and RNFL thickness values in optic nerves with and without DIR hypersignal (r = − 0.36)

Presence of DIR hypersignal in optic nerves in at least one segment lowered RNFL thickness by 17.54 μm (*p* = 0.005) in comparison to optic nerves without DIR hypersignal. Optic nerves with DIR hypersignal had significantly lower RNFL thickness values (61.06 ± 12.1 versus 82.5 ± 21.6 μm; *p* < 0.001) and significantly higher IOP values (19.8 [24–18]; versus 15.45; [18.85–13.75] mmHg; *p* = 0.008) as shown in Fig. 3. When considering all optic nerves higher IOP values could be associated with lower RNFL thickness values (r = − 0.36, *p* = 0.02) as shown in Fig. 4. The data used for this analysis is available in the supplementary material.

## Discussion

For the first time, we report on pathologic long-segment 3D DIR hypersignal in optic nerves affected by GON in glaucoma patients associated with significantly decreased RNFL thickness values and significantly increased IOP and not associated with demyelinating or ischemic optic neuropathy. It is known from a previous study that normal and healthy optic nerves always present with a hypointense signal compared to the signal intensity of ipsilateral lateral rectus muscle [23] and hypersignal on DIR is always pathologic [23].

An increase in IOP with or without associated decreased ocular perfusion and with changes in perioptic CSF pressure and protein / neurotoxic molecule content due to compartimentalization of the perioptic CSF spaces as well as additional factors (as for example patient age) might be a trigger for a cascade of events leading to damage of the RGC cells and axons with decreased RNFL thickness [8–14, 19, 24–30]. Finally a long-segment neuropathy with antegrade and retrograde axonal degeneration and disruption of axonal transport from the ONH to the lateral geniculate ganglion [5–7, 26] and with RNFL thickness loss [26–30] is the end result of the glaucomatous nerve damage. Axonal degeneration causes a hyperintense signal on T2w MR images at the nerve lesion site and distal to it [31] and thus may correspond to the long-segment DIR hypersignal observed in our patients. Most probably the diffuse T2 hyperintense signal of optic nerves described in a previous study and attributed to intraneural edema associated with acutely increased intraocular pressure may represent the same finding [32] as was obtained as DIR hypersignal in our study. The recently observed focal T2 hyperintensity of the optic nerve heads related to GON, but without associated signal intensity change of the entire nerve [33] may be part of the same imaging spectrum with DIR hypersignal of the entire nerve in GON.

Thus the clinically relevant findings of this study are that long-segment DIR hypersignal in GON should not be mistaken for the typical short-segment subclinical optic nerve demyelination usually occurring in the retrobulbar nerve segment in patients with multiple sclerosis [23].

Furthermore DIR hypersignal seems to reflect disease severity due to its association with significantly decreased RNFL thickness values and increased IOP values and may thus be an interesting clinical diagnostic tool. This should be reviewed systematically in a future study.

There are several possible restrictions to the results of our study:

1. OCT and IOP measurements were not performed on the same day as the MR examination.
2. Only selected patients were sent to MR examination in order to exclude additional pathology beside glaucoma introducing a selection bias.
3. The patient population was not homogeneous. Not only patients with open angle glaucoma, but also patients with NTG and with closed-angle glaucoma were included. This may have influenced IOP measurements.
4. Our population size is quite small as only few patients with GON are given a dedicated MRI examination. Further studies are needed to confirm our findings.
5. Every eye was evaluated separately because glaucomatous optic neuropathy can present unilaterally or bilaterally with varying degrees of severity in case of bilateral presentation. Therefore a possible correlation between both eyes in a patient potentially influencing our results was not taken into account.
6. RNFL thickness values were used as the primary marker for disease severity. However, it has to be acknowledged that other metrics such as visual field index VFI and visual field mean deviation MD are also important metrics for analysis of the disease severity. However the association between DIR hypersignal and VFI and MD was not systematically evaluated in our patients.

## Conclusion

Significantly increased IOP and significantly decreased RNFL thickness values were present in patients with glaucomatous optic neuropathy (GON) and pathologic optic nerve DIR hypersignal. DIR hypersignal seems to be a marker for disease severity in GON related to decreased RNFL thickness and may thus represent long-segment severe axonal degeneration in optic nerves in patients with GON. Venous congestion and edema within the optic nerve related to high IOP may contribute to the DIR hypersignal as well. Most importantly, long-segment DIR hypersignal in GON should not be mistaken for the typical short-segment subclinical optic nerve demyelination usually occurring in the retrobulbar nerve segment in patients with multiple sclerosis.

## Supplementary information

**Additional file 1.** contains data named “study data”. The study data contains all data used in this study, such as IOP and RNFL values.

## Data Availability

The dataset used and analysed during the current study are available from the corresponding author on reasonable request.

## Abbreviations

CI: Confidence interval
DIR: Double inversion recovery
GON: Glaucomatous optic neuropathy
IOP: Intraocular pressure
MD: Mean deficit
NTG: Normal tension glaucoma
OCT: Optical coherence tomography
ONH: Optic nerve head
RGC: Retinal ganglion cells
RNFL: Peripapillary retinal nerve fiber layer
SD: Standard deviation
